# Relationship between age and brainstem allometry in the African grasscutter (*Thryonomys swinderianus* Temminck, 1827)

**DOI:** 10.4102/jsava.v88i0.1481

**Published:** 2017-07-05

**Authors:** Chikera S. Ibe, Ekele Ikpegbu, Oliver Nzalak

**Affiliations:** 1 Department of Veterinary Anatomy, Michael Okpara University of Agriculture, Nigeria; 2 Department of Veterinary Anatomy, Ahmadu Bello University, Nigeria

## Abstract

Allometric values of brainstem structures were evaluated in African grasscutters *Thryonomys swinderianus* (*n* = 27). Brain samples were extracted from 9 animals each of 3 days (neonates), 72 days (juveniles) and 450 days of age (adults). The midbrain, pons and medulla oblongata were separated from each brain sample and dimensions and weights obtained. The weights of the midbrain in the neonate, juvenile and adult African grasscutters were 0.33 g ± 0.01 g, 0.47 g ± 0.01 g and 0.93 g ± 0.02 g, respectively. The increase from neonate to juvenile (*p* = 0.002) and adult (*p* = 0.003) was significant. The pons lengths in the neonate, juvenile and adult were 2.05 mm ± 0.05 mm, 3.86 mm ± 0.05 mm and 4.16 mm ± 0.22 mm, respectively. There was a significant increase in the length of the pons from the neonate to the juvenile (*p* = 0.002), but the increase from the juvenile to the adult period was not significant (*p* = 0.263). There was also a significant (*p* < 0.05) increase in the weights and lengths of the medulla oblongata from neonate to juvenile and adult periods. In adults, the nose-rump length and the length of the medulla were significantly negatively correlated (*r*² = 0.47; *p* = 0.043). The present study concluded that the postnatal development of some brainstem structures in the African grasscutter varies with age.

## Introduction

The African grasscutter (*Thryonomys swinderianus* Temminck, 1827), indigenous to Africa, is the fourth largest extant rodent after the porcupine, beaver and capybara (Eben 2004). Although it is a wild rodent, it is currently hunted aggressively as bushmeat, thus the attempts to breed and domesticate it are ongoing in Nigeria (Fa et al. 2002). Despite this attempt, little is known of the basic anatomy of the rodent. Knowledge of the anatomy of this rodent is essential in understanding the basis for some behavioural traits it exhibits in the wild. These traits must be taken into account in the breeding programme for efficient performance of the animal in captivity. It was in line with this that the need to study the brain anatomy of the African grasscutter arose. Also, since breeding of the rodent encompasses all developmental periods, there was a need to understand the allometric variations of the organs as the animal advances in age. Moreover, results of the study will constitute a significant addition to the increasing anatomical database of the African grasscutter.

Allometric studies are useful in understanding differential growth rates of body parts. It has also been indicated as a useful tool in the analysis of variations between ecological factors and organ shape (Sigirli & Ercan 2015), and estimation of quantitative-genetic parameters (McGuigan et al. 2010).

The brainstem was chosen for the present study due to its functional significance in the control of vital body functions such as visual reflex, respiratory, circulatory and digestive functions. It consists mainly of the midbrain, pons and medulla oblongata. As a continuation of a series of reports on the anatomy of the African grasscutter brain, the morphometric values of the brainstem structures in the neonate, juvenile and adult are hereby reported.

## Materials and methods

### Management of experimental animals

African grasscutters (*n* = 27) comprising 9 animals each of 3 days (neonates), 72 days (juveniles) and 450 days of age (adults) were used for the research. The sex of the animals was not considered. They were purchased from a grasscutter farm in Elele, Rivers State, Nigeria (4.7500° N and 6.8333° E). They were transported in ventilated wooden cages to the Veterinary Histology Laboratory of the Michael Okpara University of Agriculture, Umudike, Abia State, Nigeria. In the laboratory, they were transferred to standard laboratory animal cages and acclimatised for 1 month before the study. During the acclimatisation period, the animals were clinically examined, and confirmed to be apparently healthy. They were fed twice daily, at 8.00 am and 6.00 pm, with fresh guinea grass (*Panicum maximum*), fresh cane grass (*Eragrostis infecunda*) and commercial rodent-pelleted concentrates. Drinking water was provided *ad libitum*. The feeding troughs and drinkers were sterilised daily using sodium hypochlorite 2% w/w (Milton^®^, Rivadis, Louzy, France). The cages were also swept and disinfected daily using sodium hypochlorite 2% w/w and a-(p-nonylphenyl)-omega-hydroxylpoly (oxyethylene)-iodine complex and phosphoric acid (IOFEC-14) (Whitmoyer Laboratories Inc. USA).

### Brain extraction

All experimental animals were anaesthetised by the intramuscular administration of ketamine (Ketamine hydrochloride Injection USP, Rotex Medica, Trittau Germany) and xylazine (XYL-M 2%, VMD Livestock Pharma, Hope Maui, Arendok, Belgium). The body weight of each African grasscutter was obtained using a digital electronic balance (Citizen Scales [1] PVT Ltd., sensitivity: 0.01 g). Nose-rump lengths, tail lengths and anogenital distance were also measured using a centimetre ruler and converted to millimetres. Thereafter, each animal was placed on a dissection table in dorsal recumbency, and perfused, via the left ventricle, with 4% paraformaldehyde fixative as described by Gage, Kipke and Shan (2012). Immediately after the perfusion fixation, the head was separated from the rest of the body at the atlanto-axial joint, using a pair of scissors and knife, and the skull containing the brain was obtained after skinning and stripping off all the facial muscles (Ibe et al. 2016). Then craniotomy preceded brain extraction. Specifically, brain extraction was performed in a caudo-rostral and dorso-ventral direction, using scalpel blades, thumb forceps, rongeur and a pair of scissors. The meninges and underlying blood vessels were gently removed to expose the intact brain (Ibe et al. 2016). The mass of the whole brain was obtained using the Mettler balance P 1261 (Mettler instrument AG., Greifensee, Switzerland; sensitivity: 0.01 g).

### Separation of brainstem structures

In order to separate the brainstem from the cerebrum and cerebellum, the two cerebral hemispheres were gently pulled apart at the occipital pole to expose the *corpus callosum*. The entire *corpus callosum* together with the *septum pellucidum* and the body and rostral commissure of the fornix were severed in the midline. This completely separated the cerebrum from the brainstem and cerebellum. The cerebellum was then separated from the brainstem by severing the cerebellar peduncles. Thereafter, the brainstem was freed from cranial nerves by simple trimming. The midbrain was separated from the pons by an incision on the ventral surface of the brainstem, immediately rostral to the pons. The pons was separated from the medulla oblongata by an incision at the pontomedullary junction. The weights and dimensions of the isolated brainstem structures were obtained using the same instruments mentioned above.

### Landmark for morphometric measurements

**FIGURE 1:**
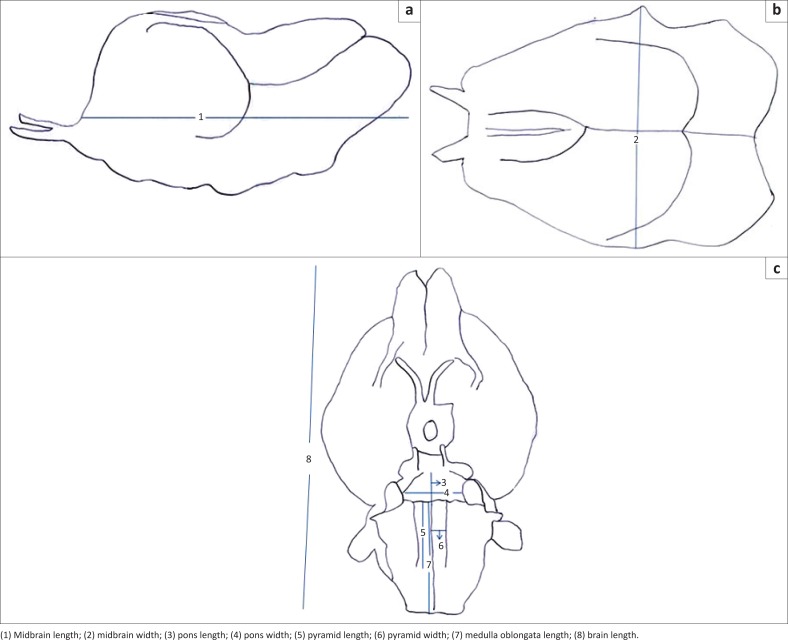
Landmark for morphometric values. (a) Lateral view of midbrain, (b) dorsal view of midbrain and (c) ventral view of the brain.

The landmarks for the measurements, as represented in Figure 1, were as follows: Brain length: rostrocaudal extent from tip of olfactory bulb to pyramidal decussation of medulla oblongata, at the level of the foramen magnum. Midbrain length: rostrocaudal extent of the lateral surface of the midbrain. Midbrain width: transverse extent of the dorsal surface of the midbrain. Pons width: extent of the pons along the width of the brain. Pons length: extent of the pons along the rostrocaudal axis of the brain. Medulla oblongata length: rostrocaudal extent from the pontomedullary junction to the pyramidal decussation. Pyramid length: rostrocaudal extent of one pyramid. Pyramid width: transverse extent of one pyramid.

### Statistical analysis of the data

Data obtained were expressed as mean ± SEM (standard error of mean), and presented in tables and graphs. Data of weight and dimensions of brainstem structures across the different postnatal stages were subjected to one-way analysis of variance, followed by Tukey’s post-hoc test to determine significance of the mean. Association between nose-rump length and dimension of brainstem structures, and between body weight and weight of brainstem structures, were determined using Pearson’s coefficient of correlation, at 95% confidence interval. Values of *p* ≤ 0.05 were considered significant. GraphPad Prism version 4 (GraphPad Software Inc., San Diego, CA) for Windows 8 was used for the statistical analysis.

## Results

The mean body weights of the neonate, juvenile and adult African grasscutters from the present study were 109.76 g ± 2.12 g, 273.39 g ± 6.70 g and 2925.56 g ± 141.96 g, respectively. The mean nose-rump lengths were 155.56 mm ± 5.85 mm, 216.89 mm ± 5.18 mm and 470.33 mm ± 8.47 mm, respectively.

The allometric values of the midbrain in the neonate, juvenile and adult African grasscutters are presented in Table 1. There was a significant increase in the weight of the midbrain from neonate to juvenile (*p* = 0.002) and adult (*p* = 0.003) periods. Similarly, there was a significant increase in the length of the midbrain from the neonate to the juvenile (*p* = 0.004) and adult (*p* = 0.001) periods. The increase in the width of the midbrain from neonate to juvenile was not significant (*p* = 0.460), but similar increase from the juvenile to adult periods was significant (*p* = 0.004).

**TABLE 1:** Age-specific allometry of the midbrain in the African grasscutter (mean ± SEM).

Midbrain parameter	Neonate (Day 3)	Juvenile (Day 72)	Adult (Day 450)
Midbrain weight (g)	0.33 ± 0.01^a^	0.47 ± 0.01^b^	0.93 ± 0.02^c^
Midbrain length (mm)	4.34 ± 0.15^a^	5.25 ± 0.10^b^	7.54 ± 0.15^c^
Midbrain width (mm)	7.21 ± 0.21^a^	7.46 ± 0.12^a^	11.50 ± 0.09^b^

Means on the same row with different alphabet superscripts are significantly different (*p* < 0.05).

Means on the same row with the same alphabet superscript are not significantly different (*p* > 0.05).

SEM, standard error of mean.

The allometric values of the pons in the neonate, juvenile and adult African grasscutters are presented in Table 2. There was a significant increase in the weight of the pons from neonate to juvenile (*p* = 0.008) and adult (*p* = 0.001) periods. Similarly, there was a significant increase in the length of the pons from the neonate to the juvenile (*p* = 0.002). However, the increase in length from the juvenile to the adult period was not significant (*p* = 0.263). Furthermore, the increase in the width of the pons from neonate to juvenile (*p* = 0.003) and adult (*p* = 0.001) periods was significant.

**TABLE 2:** Age-specific allometry of the pons in the African grasscutter (mean ± SEM).

Pons parameter	Neonate (Day 3)	Juvenile (Day 72)	Adult (Day 450)
Pons weight (g)	0.12 ± 0.01^a^	0.16 ± 0.01^b^	0.36 ± 0.01^c^
Pons length (mm)	2.05 ± 0.05^a^	3.86 ± 0.05^b^	4.16 ± 0.22^b^
Pons width (mm)	11.27 ± 0.37^a^	21.40 ± 0.19^b^	26.81± 0.35^c^

Means on the same row with different alphabet superscripts are significantly different (*p* < 0.05).

Means on the same row with the same alphabet superscript are not significantly different (*p* > 0.05).

SEM, standard error of mean.

The allometric values of the medulla oblongata in the neonate, juvenile and adult African grasscutters are presented in Table 3. There was a significant increase in the weight of the medulla oblongata from neonate to juvenile (*p* = 0.014) and adult (*p* = 0.001) periods. Similarly, there was a significant (*p* < 0.05) increase in the length and width of the pyramids from the neonate to the juvenile and adult periods.

**TABLE 3:** Age-specific allometry of the medulla oblongata in the African grasscutter (mean ± SEM).

Medulla oblongata parameter	Neonate (Day 3)	Juvenile (Day 72)	Adult (Day 450)
Medulla oblongata weight (g)	0.37 ± 0.01^a^	0.49 ± 0.01^b^	1.25 ± 0.05^c^
Medulla oblongata length (mm)	9.26 ± 0.07^a^	11.05 ± 0.06^b^	13.54 ± 0.10^c^
Pyramid length (mm)	6.91 ± 0.08^a^	7.97 ± 0.02^b^	10.36 ± 0.04^c^
Pyramid width (mm)	1.04 ± 0.04^a^	1.81 ± 0.03^b^	2.72 ± 0.13^c^

Means on the same row with different alphabet superscripts are significantly different (*p* < 0.05).

Means on the same row with the same alphabet superscript are not significantly different (*p* > 0.05).

SEM, standard error of mean.

**FIGURE 2:**
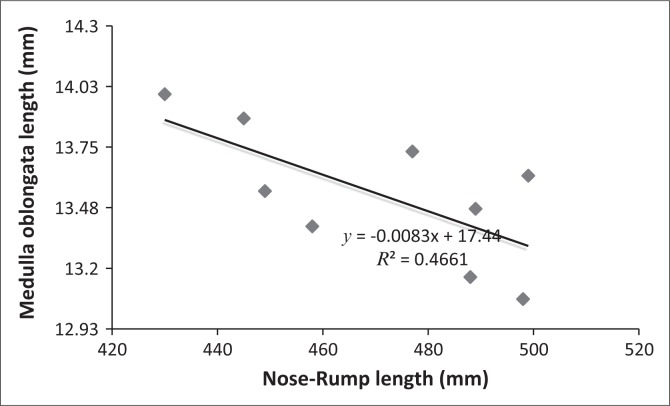
Negative linear relationship between nose-rump and medulla oblongata lengths in the African grasscutter on postnatal day 450.

There was a significant negative correlation between the nose-rump length and medulla oblongata length (*r*² = 0.47; *p* = 0.043) on postnatal day 450. The negative correlation result was subjected to regression analysis, and a regression formula was deduced on a graph as follows (Figure 2):

*y* = -0.0083x + 17.44                [Eqn 1]

where *y* = length of the medulla oblongata and x = known nose-rump length.

## Ethical considerations

The experimental protocol was approved by the Ethical Committee of Ahmadu Bello University, Zaria, Nigeria. Management of the experimental animals was as stipulated in the Guide for the Care and Use of Laboratory Animals, 8th Edition, National Research Council, USA (National Academic Press, Washington, DC: http://www.nap.edu).

## Discussion

Information on animal body mass is essential in the formulation of drug dosages and feed regimens. The body weight of an adult African grasscutter obtained from the present study (2.925 kg) is comparable to the value of 2.6 kg reported by Ajayi et al. (2011), 2.5 kg (maximum value) reported by Byanet and Dzenda (2014) and 2.6 kg reported by Hagan et al. (2016). However, body weight as high as 8.4 kg was reported by Okorafor et al. (2013). Hagan et al. (2016) found no significant difference between body masses of male and female African grasscutters and Okorafor et al. (2013) reported the absence of seasonal variation in the body weight of the animal. Similarly, the body weight of neonate African grasscutters obtained from the present study (109.76 g) is comparable to the value of 110.0 g obtained by Asibey (1981).

Gross morphometric evaluation of brain structures may be a postulate of the size and number of neurones and neuroglia in the brain, as Herculano-Houzel (2007) observed that rodents with larger brains have larger numbers of neurones. Therefore, morphometric results of brain parts have been linked to the acuity of the functions of the brain part. McDaniel (2005) and Narr et al. (2007) were in agreement that a strong correlation exists between brain size and cognition. The significant increase in the mean weight and length of the midbrain as the African grasscutter advanced in age is said to be as a result of, or may be attributed to, the increase in the size and number of neuronal and neuroglial cells (Herculano-Houzel 2007). All the morphometric values of the midbrain in the adult African grasscutter from the present result are higher than the corresponding values obtained in the adult African giant pouched rat (Ibe et al. 2010). This is in agreement with the report that an adult African grasscutter is bigger than an adult African giant pouched rat (Byanet & Dzenda 2014).

The weight of the adult medulla oblongata recorded in the present study (1.25 g) is closer to the value of 1.104 g reported for adult African grasscutters by Ajayi et al. (2011), but higher than the value of 0.570 g reported for adult African giant pouched rat by Ibe et al. (2010). Similarly, the length of the adult medulla oblongata recorded in the present study (13.54 mm) is similar to the value of 13.76 mm reported by Ajayi et al. (2011), but more than the value of 10.20 mm reported for the adult African giant pouched rat by Ibe et al. (2010). This further buttresses the report that an adult African grasscutter is bigger than an adult African giant pouched rat.

The negative linear correlation recorded between nose-rump and medulla oblongata lengths in this study implies that the length of the medulla oblongata decreased with an increase in nose-rump length in the African grasscutter. The brainstem develops earlier than higher brain centres (Woolfolk & Margetts 2013). Thus, as the medulla oblongata developed early, it may have ceased growing even as the animal continued to grow linearly in the nose-rump axis. The importance of the regression formula is that the length of the medulla oblongata of a live adult African grasscutter can be estimated from a known nose-rump length.

## Conclusion

This work expounded the relationship between brainstem structures and postnatal age in the African grasscutter and compared the findings with those of other rodents in available literature. The study revealed a significant increase in weight and dimensions of midbrain and medulla oblongata as the rodent advanced in age, from neonate, through juvenile, to adult. The study also reported a significant negative correlation between the lengths of nose-rump and medulla oblongata in the adult African grasscutter, and a regression formula was deduced. This formula can be used to estimate the length of the medulla oblongata of any adult live African grasscutter of known nose-rump length. This study has added to the growing literature on the brain anatomy of the African grasscutter, and it is expected to serve as a base-line for future studies involving the structure of the brainstem of the rodent.
